# Efficacy and safety of accelerated partial breast irradiation: a meta-analysis of published randomized studies

**DOI:** 10.18632/oncotarget.19225

**Published:** 2017-07-13

**Authors:** Gengchun Liu, Zhongyi Dong, Baqun Huang, Yuelong Liu, Yan Tang, Qing Li, Yihui Zhu

**Affiliations:** ^1^ Department of Radiation Oncology, Xiangtan City Central Hospital, Xiangtan 411100, Hunan Province, China; ^2^ Department of Radiation Oncology, Nonfan Hospital, Southern Medical University, Guangzhou 510515, Guangdong Province, China

**Keywords:** efficacy, safety, accelerated partial breast irradiation, meta-analysis

## Abstract

**Background and purpose:**

Accelerated partial breast irradiation (APBI) technology has theoretical advantages in comparison with traditional adjuvant radiation therapy (whole-breast irradiation; WBI) after breast-conserving surgery. However, published randomized controlled trials have shown inconsistent outcomes. Therefore, a comprehensive assessment of the effectiveness and safety of APBI technology is needed.

**Results:**

A total of 7 studies of 7452 patients were included in this analysis. All 7 studies reported local recurrence as an outcome. Meta-analysis of 5 trials that included 6486 patients showed significantly different 5-year local recurrence rates for APBI and WBI groups (hazard ratio = 4.54, 95% confidence interval: 1.78–11.61, *p* = 0.002). Further analysis showed that this difference may be related to the choice of treatment methods. Benefit was conferred to the APBI group for the outcome of non-breast cancer deaths. There was no significant difference between the two groups in terms of nodal recurrence, systemic recurrence, overall survival, or mortality rates. Toxicity side effects and cosmetic effects were similar in both groups, but intraoperative radiotherapy seemed to have a greater acute response.

**Material and methods:**

Searches for relevant randomized controlled trials of APBI versus WBI were performed using the following sources: PubMed, EMBASE, Cochrane Library, Web of Science. Two independent observers evaluated the identified studies. The meta-analysis was conducted using RevMan 5.2 software.

**Conclusions:**

Although the analysis showed that patients receiving APBI had a higher local recurrence rate, subgroup analyses suggested that this might be related to treatment options. Patients who receive accurate radiotherapy may have greater benefits. APBI is a promising treatment technology and more phase III clinical trials are expected based on new treatments.

## INTRODUCTION

Breast-conserving surgery followed by whole breast radiotherapy (with or without a tumor bed boost) has become a general, consensus choice of therapy for patients with early breast cancer [1–3]. Compared with breast-conserving surgery alone, breast-conserving surgery with postoperative adjuvant radiotherapy has been shown to be effective for reducing the recurrence rate in the ipsilateral breast (5-year recurrence rate: 26.0% vs. 7.0%, respectively), breast cancer-related mortality (15-year breast cancer-related mortality: 35.9% vs. 30.5%, respectively), and total mortality [4]. Compared with mastectomy, this treatment regimen is associated with similar rates of local recurrence and overall survival [5].

Although postoperative radiotherapy is an essential part of breast-conserving therapy for early-stage breast cancer, traditional whole breast radiotherapy requires 5–7 weeks of treatment [6], which results in rejection by approximately 20% of patients [7]. In the United States, this percentage has reached 10–40% [8–10]. Currently, another option is also available for patients who need postoperative radiotherapy after breast-conserving surgery: accelerated breast irradiation (APBI). This treatment only takes about 1 week, which is more likely to be accepted by patients. Besides, APBI technology is promising because of its smaller irradiation range, which is theoretically expected to reduce toxic side effects and improve cosmetic effects and quality of life [11–13].

APBI technology was introduced into clinical practice in 1990s [14, 15]; however, the medical community remains cautious about this technology. For more than 20 years, APBI has not been used widely in clinical practice. According to currently published criteria, it is only applicable to highly selected patients with breast cancer [16], while other indications are still under exploration.

In this study, we have performed a literature review and meta-analysis of controlled studies to evaluate the safety and efficacy of APBI technology. As a result of advances in radiotherapy, a variety of new radiotherapy methods have also been applied to APBI. This article also provides a preliminary discussion of these new methods.

## RESULTS

### Selection and characteristics of the studies

**Figure 1:**
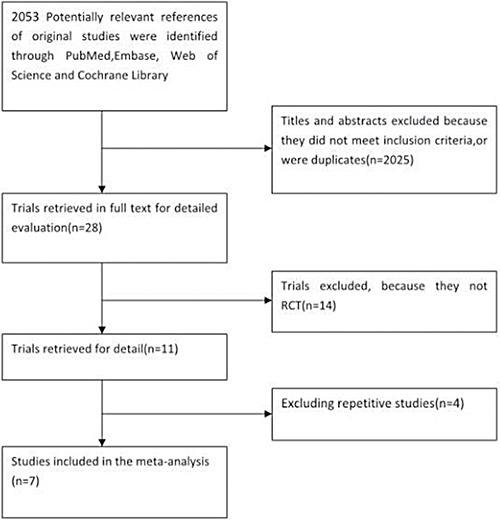
Selection of the studies

In our literature review, 2053 articles were selected as potentially relevant references, of which 2025 were excluded based on their titles and abstracts. After carefully review of the full texts of the remaining 28 papers, 14 articles on randomized controlled studies were selected [3, 15, 17–28]. Reading a study by Dodwell et al. [17] revealed that 7 patients in the APBI group received WBI, and that their treatment lasted as much as 28 days, which we viewed as incompatible with the concept of accelerated irradiation. Hence, Dodwell et al.'s study was not included as a randomized controlled trial in our meta-analysis, even though it had been included in prior meta-analyses [29–33]. After additionally excluding repetitive studies and earlier publications of the same studies, a total of 7 articles [15, 22–26, 28] that included 7452 patients were short-listed for the final meta-analysis (Figure 1). The publication dates ranged from 1993 to 2016. The characteristics of the included trials are summarized in Table 1.

**Table 1:** Characteristics of studies included in the meta-analysis

Trial	Period of inclusion	Treatment arms	Inclusion criteria	Stage	Primary end point	Follow-up duration, Median (mo)	technique of APBI
Ribero 1993	1982–1987	APBI (*n* = 353) WBI (*n* = 355)	< 70 years; tumor < 4 cm; cN0	I–II	LR; OS	7	electrons bean
Polgar 2013	1988–2004	APBI (*n* = 128) WBI (*n* = 130)	Unifocal tumor; ≤ pT2; cN0, pN0, or pN1mi	I–II	LR;DFS	10	multicatheter brachytherapy
Rodriguez 2013	-	APBI (*n* = 51) WBI (*n* = 51)	P60 years; IDC; unifocal tumor; ≤ pT2; cN0	I–II	LR;DM	5	3D-CRT
Veronesi 2013	2000–2007	APBI (*n* = 651) WBI (*n* = 654)	48–75 years; tumor < 2.5 cm	I–II	LR;RR;	5	Intraoperative
Vaidya 2014	2000–2012	APBI (*n* = 1679) WBI (*n* = 1696)	P45 years; IDC; T1–T2; unifocal tumor; no EIC	I–II	LR;RR	5	Intraoperative
Livi 2015	2005–2008	APBI (*n* = 260) WBI (*n* = 260)	> 40 years; T ≤ 2.5 cm; unifocal tumor; no EIC	I–II	LR;BCD;N-BCD	5	IMRT
Strand 2016	2004–2009	APBI (*n* = 633) WBI (*n* = 551)	≥ 45 years; T ≤ 3 cm; cN0	I–II	LR;DFS;OS;	5	multicatheter brachytherapy

Abbreviations: IDC: Invasive ductal carcinoma; EIC: Extensive intraductal carcinoma; LR: Local recurrence; RR: regional recurrences; DM: Distant metastasis; OS, overall survival; DFS: Disease-free survival; BCD: Breast cancer deaths; N-BCD: Non-breast cancer deaths; 3D-CRT: three-dimensional conformal radiotherapy; IMRT: intensity-modulated radiation therapy

### Methodological quality of the studies

**Figure 2:**
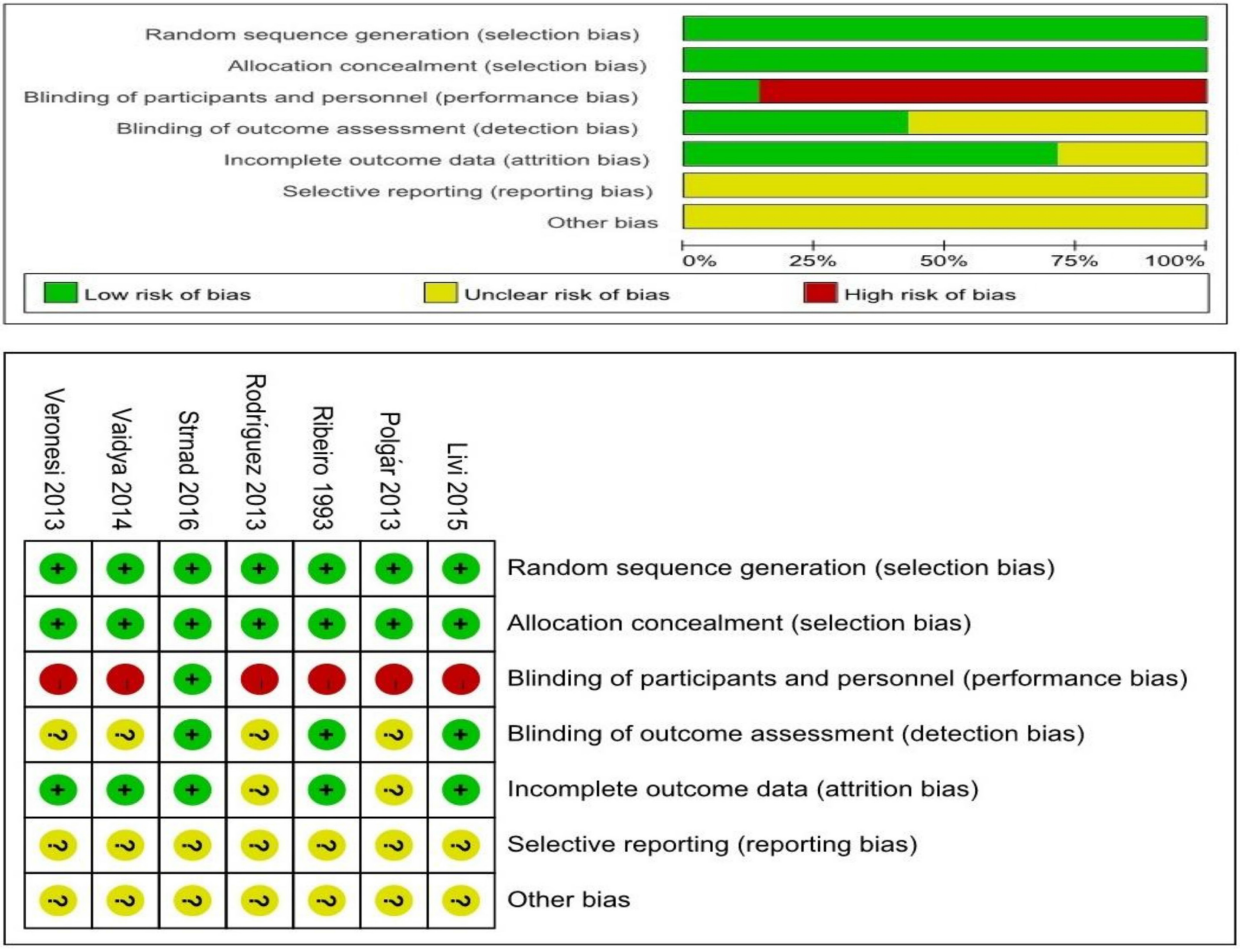
Methodological quality of the studies

Two independent observers used the Cochrane Institute's Risk of Bias table to evaluate the methodological quality of the included studies. Because it is difficult to achieve patient blinding in relevant trials, we removed blinding from the characteristics that were used to evaluate study quality. In our evaluation, 5 trials were assessed as being high quality and having a low risk of bias. The remaining 2 studies were rated as having an unclear risk of bias (Figure 2).

### Local recurrence

**Figure 3:**
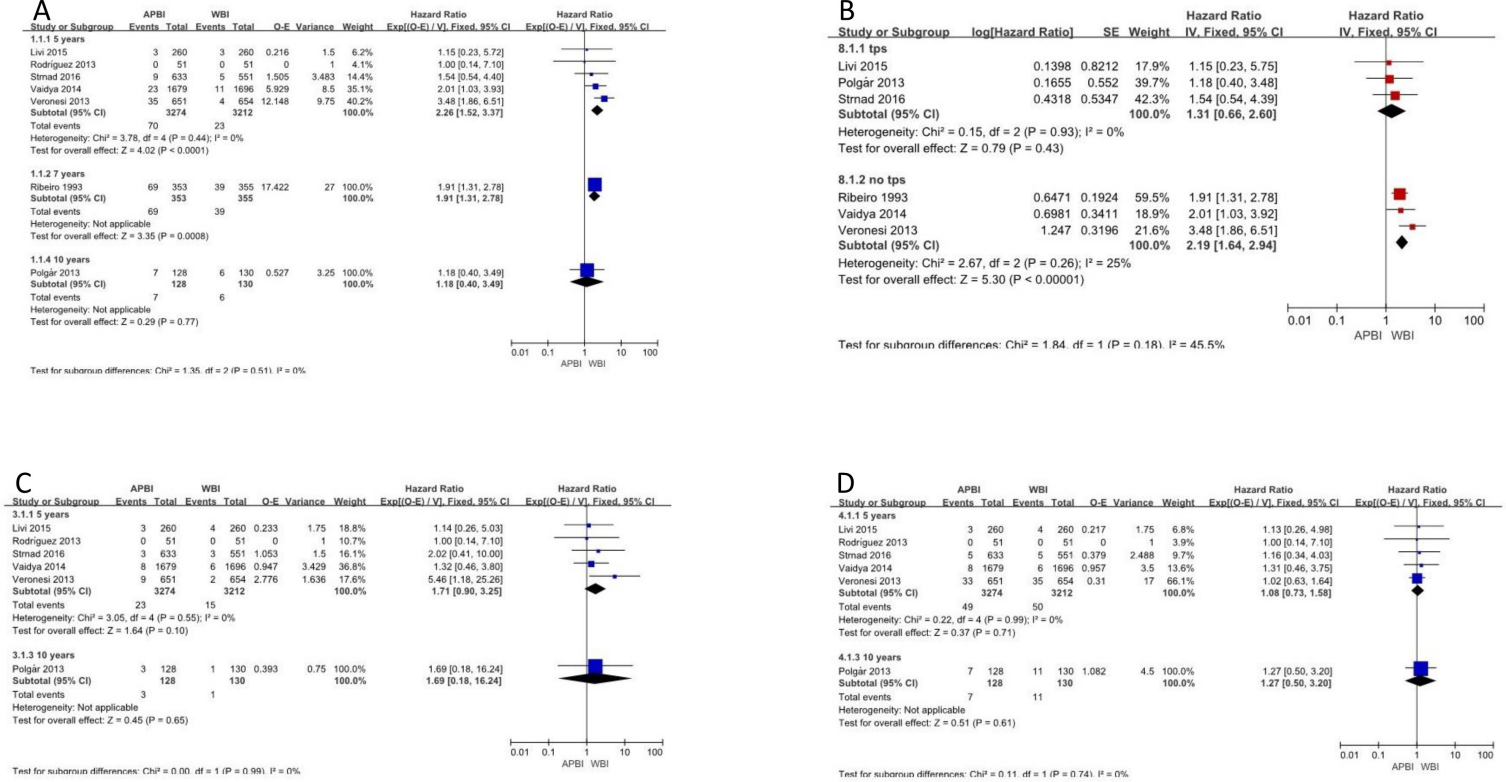
Local recurrence; nodal recurrence; systemic recurrence

Local recurrence rates were reported for all 7 studies. Five-, 7–, and 10-year local recurrence were reported by 5, 1, and 1 of the studies, respectively. Meta-analysis showed a significant benefit to 5-year local recurrence rates in whole-breast irradiation (WBI) groups (hazard ratio [HR] = 2.26, 95% confidence interval [CI]: 1.52–3.37, *p* ≤ 0.0001; p of heterogeneity = 0.44, I^2^ = 0%). Similar results were observed in a subgroup analysis of 7-year local recurrence rates (HR = 1.91, 95% CI: 1.31–2.78, *p* = 0.0008) (Figure 3A). However, there was no statistically significant difference in local recurrence rates at 10 years of follow-up.

We also performed subgroup analyses based on the treatment methods that patients received. Analysis of the subgroup of patients who received therapy with Radiotherapy Treatment Planning System (TPS) indicated no significant difference between APBI and WBI in terms of local recurrence (HR = 1.31, 95% CI: 0.66–2.60, *p* = 0.43; p of heterogeneity = 0.93, I^2^ = 0%). To account for the different follow-up times that were employed in the included studies, the generic inverse variance method was used to combine HRs for this subgroup analysis. In contrast, in the subgroup of patients who did not receive TPS, WBI showed a clear benefit (HR = 2.19, 95% CI: 1.64–2.94, *p* < 0.0001; p of heterogeneity = 0.26, I^2^ = 25%) (Figure 3B).

### Nodal recurrence

The 5 articles that reported 5-year nodal recurrence were analyzed and showed no significant difference between the APBI and WBI groups (HR = 1.71, 95% CI: 0.90–3.25, *p* = 0.08; p of heterogeneity = 0.55, I^2^ = 0%). Further, no significant difference was found for 10-year nodal recurrence (Figure 3C).

### Systemic recurrence

The outcome of systemic recurrence was reported in 6 studies. Meta-analysis of the results revealed no significant benefit in favor of APBI or WBI for any subgroups or outcome evaluation times. The HRs for systemic recurrence at 5 and 10 years were 1.08 (95% CI: 0.73–1.58) and 1.27 (95% CI: 0.50–3.20), respectively (Figure 3D).

### Disease- specific survival

**Figure 4:**
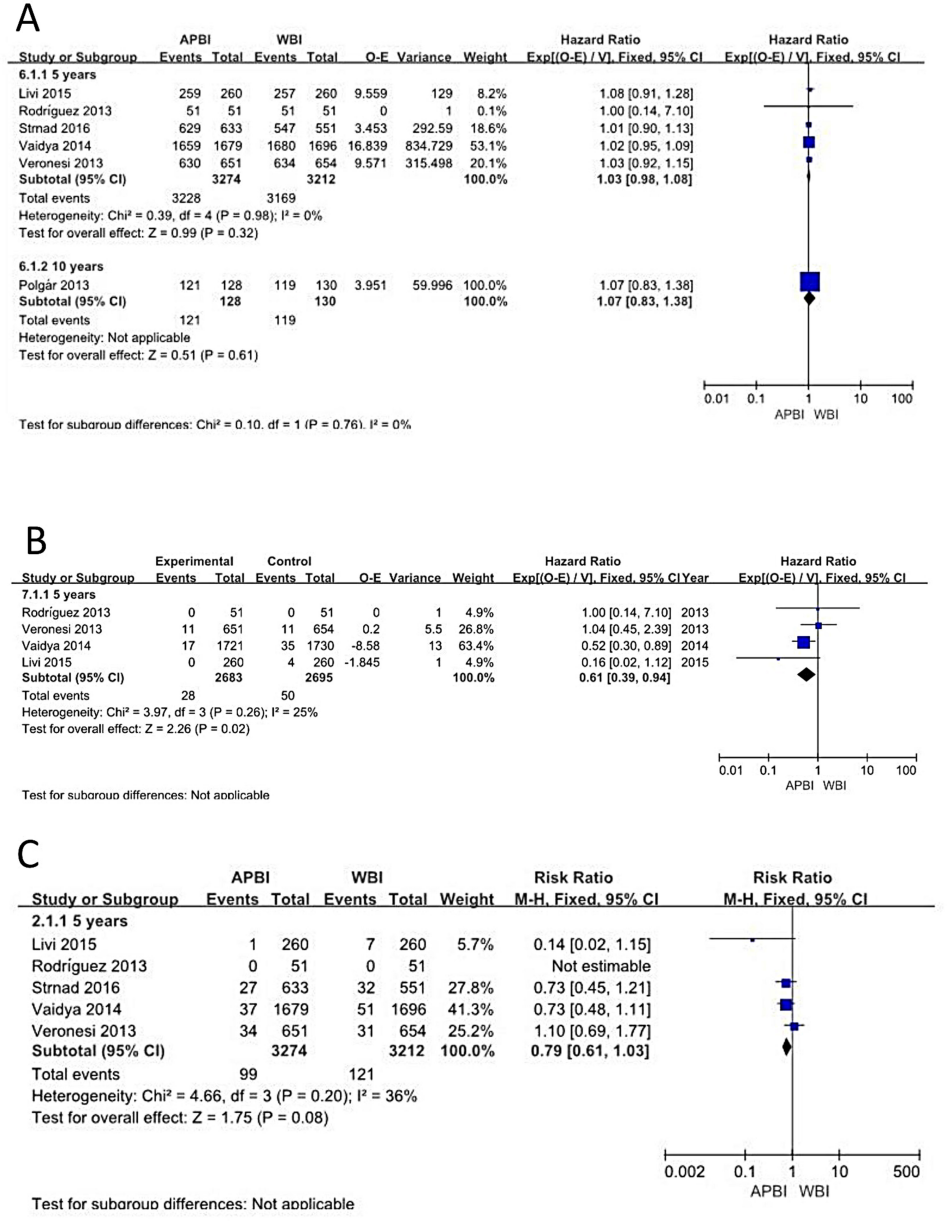
Disease-specific survival; Non-breast cancer deaths; Mortality

Disease-specific survival was reported for 6 of the studies that were eligible for our meta-analysis. The analysis showed that patients who received APBI had equivalent disease-free survival to those who received WBI (HR = 1.03; 95% CI = 0.98–1.09; *p* = 0.18. There was no statistically significant difference in disease-free survival between the APBI and WBI groups (Figure 4A).

### Non-breast cancer deaths

Regarding non-breast cancer deaths, analysis of the 4 studies showed that the APBI group benefited significantly more than the WBI group at 5 years of follow-up (HR = 0.61, 95% CI: 0.39–0.94, *p* = 0.02; p of heterogeneity = 0.26, I^2^ = 25%). However, there was no significant difference in outcomes between the 2 groups at 10 years (Figure 4B).

### Mortality

Mortality was reported for 6 studies with a total of 6600 patients. Meta-analysis showed no statistically significant difference between the APBI and WBI groups (HR = 0.79; 95% CI = 0.61–1.03; *p* = 0.08; p of heterogeneity = 0.20; I^2^ = 36%), but the APBI group appeared to show a non-significant trend towards greater benefit (Figure 4C).

### Toxicity, quality of life, and cosmetic effects

Seven studies reported skin side effects and cosmetic effects. Because the endpoint and standards that they used were not uniform, we have only presented a descriptive analysis of their findings (Table 2).

**Table 2:** Toxicity, quality of life, and cosmetic effects

Study	Group	Acute skin toxicity Grade ≥ 2	Late skin toxicity Grade ≥ 2	Physician-rated cosmetics excellent	Subcutaneous tissue	Grade 3 fibrosis	Grade 2–3 breast pain	Fat necrosis	Breast oedema	Rid fractures
Ribero 1993	APBI		14%					5%	2%	2%
	WBI		15%					1%	4%	1%
Polgar 2013	APBI			28.8%						
	WBI			16.4%						
Rodriguez 2013	APBI	17.6% (*p* ≤ 0.0001)	0	> 75%						
	WBI	74.5%	0	> 84%						
Veronesi 2013	APBI	1.08%	1.29%					4.74% (*p* = 0.04)		
	WBI	7.77%	1.21%					2.43%		
Vaidya 2014	APBI									

## DISCUSSION

APBI technology is based on the following 2 findings: First, a number of studies have shown that about 80.0% of breast recurrence lesions are located around the incision site [34–36]. Vicini et al. [37] found that only 9.0% of patients with breast cancer underwent resection of extramural residual tumor at 1.5 cm or greater from the margins of the original resection. Therefore, partial breast irradiation (PBI) is sufficient. Because the irradiation range is narrowed, accelerated large-division irradiation does not result in acute or late radiotherapy responses. PBI further reduces the radiation doses to the heart, lung, chest wall, and contralateral breast, as well as to the ipsilateral mammary gland. These reductions make it possible for breast-conserving surgery and radiation therapy to be applied for patients who underwent breast-conserving surgery and subsequently developed recurrence.

Second, the α/β ratio of breast cancer cells is generally considered to be 3–4 [38–40]. For such tumors, prolonged treatment will not provide any benefit to efficacy. On the other hand, shortened treatment regimens will reduce the economic burdens that are faced by patients, improve patients’ adherence to treatment, and eliminate problems with delays in radiotherapy or chemotherapy. In fact, when postoperative radiotherapy is delayed for 20 to 26 weeks, disease-related mortality is increased [41].

Although APBI technology has many advantages, controversy remains regarding its indications. At present, there are 4 major published standards: those of ASTRO (American Society for Radiation Oncology), GECESTRO (Grouped European de Curietherapie-European Society for Radiotherapy and Oncology), ABS (American Brachytherapy Society), and ASBS (American Society of Breast Surgeons). In addition to consistent standards regarding tumor size and negative margins, other criteria may vary across the criteria, including for age, molecular typing, lymph node invasion, and other characteristics. As more results from evidence-based medicine are published, the standards are iteratively updated. In 2017, ASTRO reduced the minimum recommended age for APBI from 60 years to 50 years [42].

We reviewed several previously published meta-analysis of APBI. With regard to nodal recurrence, systemic recurrence, and overall survival, the individual analyses were fairly consistent in their findings, tending to indicate that there was no significant difference between APBI and WBI groups. The main controversy concerns local recurrence. Valachis et al. [33] and Ye et al. [32] arrived at opposite conclusions regarding local recurrence. However, their meta-analyses included only 3 and 4 studies, respectively, and we believe that the limited sample sizes affect the objectivity of the results. Kong and colleagues [29] found that patients who received APBI had a higher rate of local recurrence than did patients who received WBI (OR = 1.54; 95% CI: 1.15–2.06; *p* = 0.004). The analysis of Kong and colleagues incorporated 10 studies, including both randomized controlled trials and non-randomized controlled studies. All of the abovementioned studies used odds ratios (ORs) in their meta-analyses. However, because HRs are natural indicators for time-to-event data, we believe that they are more accurate than ORs or relative risks (RRs) for analyses of survival. Therefore, we used HRs in our meta-analysis.

Marta et al. [30] carried out a subgroup analysis of local recurrence in 6 studies and observed that their results, “showed significant benefit in the WBI versus APBI group with respect to 5-year local recurrence.” Unfortunately, Marta et al. incorrectly used the same data from 2 studies (Ribeiro et al. [15] and Polgar et al. [22]), and also included Dodwell et al.'s study [17], which we do not think is a standard controlled trial of APBI. Further, the z-score that Marta et al. used is slightly different from that found in a normal probability table.

The most recent meta-analysis was performed by Vaidya and colleagues [31], who included 9 randomized controlled trials. However, Vaidya and colleagues only analyzed breast-cancer-related mortality, ignoring local recurrence, nodal recurrence, systemic recurrence, and overall survival..

None of the abovementioned meta-analyses agreed about local recurrence rates. Marta et al. and Valachis et al. [33] argued that APBI increases the local recurrence rate, whereas Ye et al. [32] and Kong et al. [29] reached the opposite conclusion. Vaidya and colleagues [31] did not analyze local recurrence.

The critical difference between APBI and WBI is the range of irradiation. We believe that comparing tumor bed relapse is not sufficient to rigorously evaluate whether patients benefit from extended exposure. Therefore, local recurrence was defined as recurrence in the ipsilateral breast (including in the same or different quadrants) in our meta-analysis. We included 7 studies that reported local recurrence and found significant differences in local recurrence rates at 5 years (HR = 2.33, 95% CI: 1.45–3.74, *p* = 0.0005; p of heterogeneity = 0.17, I^2^ = 36%) and 7 years (HR = 1.91, 95% CI: 1.30–2.79, *p* = 0.0009; p of heterogeneity = 0.60, I^2^ = 0%). We also noted that the choice of treatment technique had an effect on the analysis. Ribeiro et al.'s research [15], which was conducted more than 20 years ago, used single electron beam irradiation. Vaidya et al. [25] and Veronesi et al. [24] investigated treatment with intraoperative radiotherapy, using Intraoperative Electron Radiation Therapy (IOERT)and Targeted intra-operative radiotherapy (TARGIT) treatments, respectively. Obviously, these 2 treatment methods did not involve accurate definitions of the target area or dose distribution. In contrast, three-dimensional conformal, intensity-modulated radiotherapy, or brachytherapy based on TPSs are more accurate in terms of target volumes and doses. Therefore, we performed a subgroup analysis of treatment techniques. We observed no significant difference between APBI and WBI in the TPS subgroup (HR = 1.31, 95% CI: 0.66–2.60, *p* = 0.43; p of heterogeneity = 0.93, I^2^ = 0%). On the other hand, WBI was shown to confer clear benefit in the no-TPS subgroup (HR = 2.19, 95% CI: 1.64–2.94, *p* < 0.0001; p of heterogeneity = 0.26, I^2^ = 25%). We therefore concluded that, although APBI showed significant disadvantages at 5 and 7 years (in resemblance with the findings of previous meta-analyses), the selection of appropriate treatment techniques might eliminate this difference. However, to support this conclusion, longer follow-up times and more clinical trials are needed to provide a higher level of evidence. Evermore new technologies have been applied to APBI, such as image-guided radiotherapy and Tomotherapy and have achieved the desired results. However, there have not been any randomized controlled studies of these technologies, so their effectiveness and safety remain to be confirmed. Perhaps it is time to consider the introduction of new technologies to replace the old technologies that have been used for APBI.

There are no significant differences between APBI and WBI in terms of systemic recurrence, overall survival, or mortality, which indicates that local recurrence may not be a cause of distant metastasis or death, as is suggested by Fisher and colleagues’ research [43]. However, we also noted that most patients with relapses underwent salvage surgery, which effectively improves survival. Therefore, local recurrence may actually be associated with mortality, but salvage surgery may have obscured this association.

At the same time, it is interesting to note that APBI showed a benefit for the outcome of non-breast cancer death in out meta-analysis (HR = 0.61, 95% CI: 0.39–0.94, *p* = 0.02; p of heterogeneity = 0.26, I^2^ = 25%). This finding might be explained by the reduced irradiation that is offered by APBI, which may have translated to better protection of organs at risk, including the heart and lungs. The main heterogeneity in this finding is contributed by Vaidya et al.'s [25] study. Once we removed their study, we found no significant difference between the APBI and WBI groups. This may suggest that, although the intraoperative radiotherapy presents a higher local recurrence rate, it is the best way to protect organs at risk. However, the number of studies included in our meta-analysis is small, and there is no specific reason for non-breast cancer deaths to have been reduced by APBI. Therefore, this conclusion needs further confirmation.

Some non-radiological factors also play an important role in non-breast cancer deaths. Mell and colleagues’ [44] study shows that age, race, and comorbidity are also relevant factors. They have used the following formula to obtain risk scores: (0.06 × Age + 0.56 × Black race + 0.66 × Comorbidity - 2.4) × 100/4.16. Scores equal to or higher than 39.4 indicate membership in the high-risk group, and lower scores indicate membership in the low-risk group. For high-risk patients, there should be an increased focus on prevention of non-breast cancer death. The relationship between this subgroup and APBI is worthy of further study.

Regarding acute skin toxicity, APBI showed a greater advantage. Acute skin toxicity was significantly lower in the APBI group than in the WBI group in all 4 studies that reported acute skin reactions. In terms of late skin toxicity and physician-rated cosmesis, there was no significant difference between the 2 groups in most of the reports. Ribeiro et al. [15] reported a higher probability of late skin toxicity in the APBI group, which we believe is related to the use of electron beam therapy. Veronesi et al. [24] and Ribeiro et al. [15] reported fat necrosis, which was significantly more common in the APBI group than in the WBI group. We think that these findings are related to the use of no TPS in either trial, which could have led to high rates of normal breast tissue surrounding the tumor bed, because of the inability to accurately calculate irradiation volumes and doses. The incidence of breast pain was lower in the APBI group in both trials. Overall, there was no significant difference between the 2 groups with respect to radiotoxic side effects or cosmetic effects.

Blinding of patients and participants was not possible because there are large differences in times and treatment methods for APBI and WBI. This is the main limitation of our meta-analysis. Two of the included articles [24, 28] clearly expressed that patients, clinicians, and investigators were all aware of treatment arm assignments. In addition, most studies did not have an independent staff for data analyses. Indeed, only Vaidya and colleagues [25] used an independent senior clinician for the outcome assessment. However, we believe that the lack of investigator blinding in these experiments will not have much of an effect on the results.

In conclusion, among patients who had received breast-conserving treatment for early breast cancer, the rate of local recurrence was significantly higher for APBI than for WBI. However, non-breast cancer deaths were less common for APBI than for WBI. There were no statistically significant differences in nodal recurrence, systemic recurrence, overall survival, or mortality rates between APBI and WBI groups. The incidence of acute skin toxicity was lower in the APBI groups, but no significant differences were observed for later skin toxicity or cosmetic effect. At the same time, we believe that careful patient selection and treatment selection are equally important. Based on our preliminary investigation of treatment technologies, we also recommend that TPSs should be employed, that electron beam technology should be eliminated, and that intraoperative radiotherapy should be used with caution.

## MATERIALS AND METHODS

### Literature search

Searches were performed using PubMed (1966-December 2016), the Cochrane Library (Issue 3, 2008), EMBASE (1974-December 2016), and Web of Science (1974-December 2016). The selected search terms included APBI, PBI, accelerated partial, accelerated partial irradiation, accelerated partial breast irradiation, interstitial brachytherapy, multicatheter interstitial brachytherapy (MIB), balloon catheter brachytherapy, intracavitary brachytherapy, intraoperative radiotherapy (IORT), conformal external beam, three-dimensional conformal radiotherapy (3D-CRT), intensity-modulated radiotherapy (IMRT), breast cancer, breast neoplasm, breast tumor, human mammary carcinoma, human mammary neoplasm, clinical trial phase III, and randomized controlled trials. Other relevant studies and publications were also reviewed.

### Inclusion and exclusion criteria

All studies were reviewed by 2 independent reviewers (GCL and ZYD), and discrepancies were resolved by a third reviewer (BQH). We reviewed all studies of WBI and APBI for early breast cancer after breast-conserving surgery. Randomized controlled studies were included in this analysis, regardless of whether they were blinded and their sample sizes. The exclusion criteria were as follows: 1) non-randomized controlled trials, single arm studies, meeting abstracts, case reports, and trail designs; 2) articles in languages other than English; and 3) non-human research. The primary endpoint for our analysis was local recurrence, which we defined the presence of the any of the following: recurrence in the ipsilateral breast (including in the same or different quadrants), nodal recurrence, systemic recurrence, disease-specific survival, or death from any cause. Secondary outcome measures included toxic side effect and cosmetic results.

### Data extraction and quality assessment

The data from the studies were extracted by two authors (QL and YHZ) independently, and disagreements were resolved by consensus. The characteristics that were extracted from the individuals studies and articles included author, sample size, year of publication, inclusion criteria, stage, estrogen receptor or HER-2 positive status, irradiation therapy, and adjuvant systemic treatments. To evaluate the quality of the randomized controlled trials, we used the Assessing Risk of Bias Table that is recommended by the Cochrane Handbook 5.0.2. The evaluation criteria included 1) randomization method, 2) allocation concealment, 3) study blinding, and 4) presence or absence of loss to follow-up.

### Statistical analysis

Review Manager 5.2 was used for our analysis. Expected events (O-E) and log-rank variance (V) were used to estimate the HRs and 95% CIs. Sample sizes and *p*-values were obtained from the individual articles. O-E, V, and z-scores were calculated based on the method provided by Tierney et al. [45]. LogHR and seLogHR were calculated using the Review Manager 5.2 Calculator. Heterogeneity was evaluated using the Chi-square test and I^2^ test. Funnel plots were used as a check for publication bias.

## References

[1] Fisher B, Anderson S, Bryant J, Margolese RG, Deutsch M, Fisher ER, Jeong JH, Wolmark N (2002). Twenty-year follow-up of a randomized trial comparing total mastectomy, lumpectomy, and lumpectomy plus irradiation for the treatment of invasive breast cancer. New England Journal of Medicine.

[2] Morrow M (2002). Rational local therapy for breast cancer. New England Journal of Medicine.

[3] Veronesi U, Cascinelli N, Mariani L, Greco M, Saccozzi R, Luini A, Aguilar M, Marubini E (2002). Twenty-year follow-up of a randomized study comparing breast-conserving surgery with radical mastectomy for early breast cancer. New England Journal of Medicine.

[4] Clarke M, Collins R, Darby S, Davies C, Elphinstone P, Evans V, Godwin J, Gray R, Hicks C, James S, MacKinnon E, McGale P, McHugh T (2005). Early Breast Cancer Trialists’ Collaborative G Effects of radiotherapy and of differences in the extent of surgery for early breast cancer on local recurrence and 15-year survival: an overview of the randomised trials. The Lancet.

[5] Dongen JA (2000). Long-Term Results of a Randomized Trial Comparing Breast-Conserving Therapy With Mastectomy: European Organization for Research and Treatment of Cancer 10801 Trial. Journal of the National Cancer Institute.

[6] Haviland JS, Owen JR, Dewar JA, Agrawal RK, Barrett J, Barrett-Lee PJ, Dobbs HJ, Hopwood P, Lawton PA, Magee BJ, Mills J, Simmons S, Sydenham MA (2013). The UK Standardisation of Breast Radiotherapy (START) trials of radiotherapy hypofractionation for treatment of early breast cancer: 10-year follow-up results of two randomised controlled trials. The Lancet oncology.

[7] Malin JL, Schuster MA, Kahn KA, Brook RH (2002). Quality of breast cancer care: what do we know?. Journal of Clinical Oncology.

[8] Hershman DL, Buono D, Mcbride RB, Tsai WY, Joseph KA, Grann VR, Jacobson JS (2008). Surgeon characteristics and receipt of adjuvant radiotherapy in women with breast cancer. Journal of the National Cancer Institute.

[9] Voti L, Richardson LC, Reis I, Fleming LE, Mackinnon J, Coebergh JW (2006). The effect of race/ethnicity and insurance in the administration of standard therapy for local breast cancer in Florida. Breast Cancer Research and Treatment.

[10] Pawlik TM, Buchholz TA, Kuerer HM (2004). The biologic rationale for and emerging role of accelerated partial breast irradiation for breast cancer. Journal of the American College of Surgeons.

[11] Mccarthy EP, Long HN, Roetzheim RG, Chirikos TN, Li D, Drews RE, Iezzoni LI (2006). Disparities in Breast Cancer Treatment and Survival for Women with Disabilities. Annals of Internal Medicine.

[12] Vicini FA, Arthur DW (2005). Breast brachytherapy: North American experience. Seminars in Radiation Onchology.

[13] Brabender J, Vallböhmer D, Ling FC, Hoffmann AC, Lurje G, Bollschweiler E, Hölscher AH, Schneider PM, Metzger R (2008). Noncompliance with adjuvant radiation, chemotherapy, or hormonal therapy in breast cancer patients. American Journal of Surgery.

[14] Ribeiro GG, Dunn G, Swindell R, Harris M, Banerjee SS (1990). Conservation of the breast using two different radiotherapy techniques: interim report of a clinical trial. Clinical Oncology.

[15] Ribeiro GG, Magee B, Swindell R, Harris M, Banerjee SS (1993). The christie hospital breast conservation trial: An update at 8 years from inception - Clinical Oncology. Clinical Oncology.

[16] Shaitelman SF, Khan AJ, Woodward WA, Arthur DW, Cuttino LW, Bloom ES, Shah C, Freedman GM, Wilkinson JB, Babiera GV (2014). Shortened radiation therapy schedules for early-stage breast cancer: a review of hypofractionated whole-breast irradiation and accelerated partial breast irradiation. Breast Journal.

[17] Dodwell DJ, Dyker K, Brown J, Hawkins K, Cohen D, Stead M, Ash DA (2005). Randomised Study of Whole-breast vs Tumour-bed Irradiation After Local Excision and Axillary Dissection for Early Breast Cancer. Clinical Oncology.

[18] Polgar C, Fodor J, Major T, Nemeth G, Lovey K, Orosz Z, Sulyok Z, Takacsi-Nagy Z, Kasler M (2007). Breast-conserving treatment with partial or whole breast irradiation for low-risk invasive breast carcinoma--5-year results of a randomized trial. International Journal of Radiation Oncology Biology Physics.

[19] Livi L, Buonamici FB, Simontacchi G, Scotti V, Fambrini M, Compagnucci A, Paiar F, Scoccianti S, Pallotta S, Detti B, Agresti B, Talamonti C, Mangoni M (2010). Accelerated partial breast irradiation with IMRT: new technical approach and interim analysis of acute toxicity in a phase III randomized clinical trial. International Journal of Radiation Oncology Biology Physics.

[20] Vaidya JS, Joseph DJ, Tobias JS, Bulsara M, Wenz F, Saunders C, Alvarado M, Flyger HL, Massarut S, Eiermann W, Keshtgar M, Dewar J, Kraus-Tiefenbacher U (2010). Targeted intraoperative radiotherapy versus whole breast radiotherapy for breast cancer (TARGIT-A trial): an international, prospective, randomised, non-inferiority phase 3 trial. The Lancet.

[21] Polgar C, Major T, Fodor J, Sulyok Z, Somogyi A, Lovey K, Nemeth G, Kasler M (2010). Accelerated partial-breast irradiation using high-dose-rate interstitial brachytherapy: 12-year update of a prospective clinical study. Radiotherapy & Oncology.

[22] Polgar C, Fodor J, Major T, Sulyok Z, Kasler M (2013). Breast-conserving therapy with partial or whole breast irradiation: ten-year results of the Budapest randomized trial. Radiother Oncol.

[23] Rodriguez N, Sanz X, Dengra J, Foro P, Membrive I, Reig A, Quera J, Fernandez-Velilla E, Pera O, Lio J, Lozano J, Algara M (2013). Five-year outcomes, cosmesis, and toxicity with 3-dimensional conformal external beam radiation therapy to deliver accelerated partial breast irradiation. International Journal of Radiation Oncology Biology Physics.

[24] Veronesi U, Orecchia R, Maisonneuve P, Viale G, Rotmensz N, Sangalli C, Luini A, Veronesi P, Galimberti V, Zurrida S, Leonardi MC, Lazzari R, Cattani F (2013). Intraoperative radiotherapy versus external radiotherapy for early breast cancer (ELIOT): a randomised controlled equivalence trial. The Lancet Oncology.

[25] Vaidya JS, Wenz F, Bulsara M, Tobias JS, Joseph DJ, Keshtgar M, Flyger HL, Massarut S, Alvarado M, Saunders C, Eiermann W, Metaxas M, Sperk E (2014). Risk-adapted targeted intraoperative radiotherapy versus whole-breast radiotherapy for breast cancer: 5-year results for local control and overall survival from the TARGIT-A randomised trial. The Lancet.

[26] Livi L, Meattini I, Marrazzo L, Simontacchi G, Pallotta S, Saieva C, Paiar F, Scotti V, De Luca Cardillo C, Bastiani P, Orzalesi L, Casella D, Sanchez L (2015). Accelerated partial breast irradiation using intensity-modulated radiotherapy versus whole breast irradiation: 5-year survival analysis of a phase 3 randomised controlled trial. European Journal of Cancer.

[27] Meattini I, Saieva C, Marrazzo L, Di Brina L, Pallotta S, Mangoni M, Meacci F, Bendinelli B, Francolini G, Desideri I, De Luca Cardillo C, Scotti V, Furfaro IF Accelerated partial breast irradiation using intensity-modulated radiotherapy technique compared to whole breast irradiation for patients aged 70 years or older: subgroup analysis from a randomized phase 3 trial. Breast Cancer Research & Treatment.

[28] Strnad V, Ott OJ, Hildebrandt G, Kauer-Dorner D, Knauerhase H, Major T, Lyczek J, Guinot JL, Dunst J, Miguelez CG, Slampa P, Allgäuer M, Lössl K (2016). 5-year results of accelerated partial breast irradiation using sole interstitial multicatheter brachytherapy versus whole-breast irradiation with boost after breast-conserving surgery for low-risk invasive and in-situ carcinoma of the female breast: a randomised, phase 3, non-inferiority trial. The Lancet.

[29] Kong L, Cheng J, Ding X, Li B, Zhang J, Li H, Huang W, Zhou T, Sun H (2014). Efficacy and safety of accelerated partial breast irradiation after breast-conserving surgery: a meta-analysis of published comparative studies. Breast Journal.

[30] Marta GN, Macedo CR, Carvalho Hde A, Hanna SA, da Silva JL, Riera R (2015). Accelerated partial irradiation for breast cancer: systematic review and meta-analysis of 8653 women in eight randomized trials. Radiotherapy & Oncology.

[31] Vaidya JS, Bulsara M, Wenz F, Coombs N, Singer J, Ebbs S, Massarut S, Saunders C, Douek M, Williams NR, Joseph D, Tobias JS, Baum M (2016). Reduced Mortality With Partial-Breast Irradiation for Early Breast Cancer: A Meta-Analysis of Randomized Trials. International Journal of Radiation Oncology Biology Physics.

[32] Ye XP, Bao S, Guo LY, Wang XH, Ma YP, Zhang W, Wang CH, Zhang YF, Zhi F, Gao Y, Tian JH, Li R, Gao HM (2013). Accelerated Partial Breast Irradiation for Breast Cancer: A Meta-Analysis. Translational Oncology.

[33] Valachis A, Mauri D, Polyzos NP, Mavroudis D, Georgoulias V, Casazza G (2010). Partial breast irradiation or whole breast radiotherapy for early breast cancer: a meta-analysis of randomized controlled trials. Breast Journal.

[34] Fowble B, Solin LJ, Schultz DJ, Rubenstein J, Goodman RL (1990). Breast recurrence following conservative surgery and radiation: patterns of failure, prognosis, and pathologic findings from mastectomy specimens with implications for treatment. International Journal of Radiation Oncology Biology Physics.

[35] Boyages J, Recht A, Connolly JL, Schnitt SJ, Gelman R, Kooy H, Love S, Osteen RT, Cady B, Silver B, Harris JR (1990). Early breast cancer: predictors of breast recurrence for patients treated with conservative surgery and radiation therapy. Radiotherapy & Oncology.

[36] Veronesi U, Marubini E, Mariani L, Galimberti V, Luini A, Veronesi P, Salvadori B, Zucali R (2001). Radiotherapy after breast-conserving surgery in small breast carcinoma: long-term results of a randomized trial. Annals of Oncology Official Journal of the European Society for Medical Oncology.

[37] Vicini FA, Kestin LL, Goldstein NS (2004). Defining the clinical target volume for patients with early-stage breast cancer treated with lumpectomy and accelerated partial breast irradiation: a pathologic analysis. International Journal of Radiation Oncology Biology Physics.

[38] Merino T, Tran WT, Czarnota GJ (2015). Re-irradiation for locally recurrent refractory breast cancer. Oncotarget.

[39] Aly MM, Abo-Madyan Y, Jahnke L, Wenz F, Glatting G (2016). Comparison of breast sequential and simultaneous integrated boost using the biologically effective dose volume histogram (BEDVH). Radiotherapy & Oncology.

[40] Shaitelman SF (2013). Phase 2 Study of Pre-Excision Single-Dose Intraoperative Radiation Therapy for Early-Stage Breast Cancers: Six-Year Update With Application of the ASTRO Accelerated Partial Breast Irradiation Consensus Statement Criteria: VanderWalde NA, Jones EL, Kimple. Breast Diseases A Year Book Quarterly.

[41] Mikeljevic JS, Haward R, Johnston C, Crellin A, Dodwell D, Jones A, Pisani P, Forman D (2004). Trends in postoperative radiotherapy delay and the effect on survival in breast cancer patients treated with conservation surgery. British Journal of Cancer.

[42] Correa C, Harris EE, Leonardi MC, Smith BD, Taghian AG, Thompson AM, White J, Harris JR (2016). Accelerated Partial Breast Irradiation: Executive summary for the update of an ASTRO Evidence-Based Consensus Statement. Practical Radiation Oncology.

[43] Fisher B, Anderson S, Fisher ER, Redmond C, Wickerham DL, Wolmark N, Mamounas EP, Deutsch M, Margolese R (1991). Significance of ipsilateral breast tumour recurrence after lumpectomy. The Lancet.

[44] Mell LK, Jeong JH, Nichols MA, Polite BN, Weichselbaum RR, Chmura SJ (2010). Predictors of competing mortality in early breast cancer. Cancer.

[45] Tierney JF, Stewart LA, Ghersi D, Burdett S, Sydes MR (2007). Practical methods for incorporating summary time-to-event data into meta-analysis. Trials.

